# Assessment of the Physical Activity of Children with Asthma Bronchiale

**DOI:** 10.3390/sports12040114

**Published:** 2024-04-22

**Authors:** Ildikó Balatoni, Tímea Kiss, György Balla, Ágnes Papp, László Csernoch

**Affiliations:** 1 Clinical Center, University of Debrecen, 4032 Debrecen, Hungary; kiss.timea@med.unideb.hu; 2 Department of Pediatrics, Faculty of Medicine, University of Debrecen, 4032 Debrecen, Hungary; balla@med.unideb.hu (G.B.); papp.agnes@med.unideb.hu (Á.P.); 3 Department of Physiology, Faculty of Medicine, University of Debrecen, 4032 Debrecen, Hungary; csl@edu.unideb.hu

**Keywords:** physical activity, childhood, asthma, parents’ opinion, quality of life

## Abstract

Physical activity is an especially important part of everyday life for children with chronic diseases. The aim of the study was to show whether asthma is a barrier to physical activity in our society. The correlations between the severity of the disease, body mass index, and physical activity were analyzed, and parents’ opinions on whether children should participate in active sports were assessed. Physical activity of children with asthma was analyzed by questionnaires; 93 parents and their 93 children were involved in the survey. The age of children was 12.6 ± 3.5 years (mean ± SD), 69.9% were boys, 30.1% were girls. A total of 93.4% of the respondents participated in a physical education program and 56.5% also attended sporting activities on a regular basis. In terms of disease severity, 61.2% of the children had mild asthma, 37.6% moderate, and 1.2% severe, and 6.5% of the respondents also stated that their children’s illness had been consistently or frequently limiting their performance concerning their school or home duties over the past four weeks. Of the parents surveyed, 12% felt that physical activity was not appropriate in the context of this disease. We concluded that fear of the consequences of physical activity depends largely on education, which should involve parents, teachers, and coaches.

## 1. Introduction

Physical activity and healthy lifestyle are important elements of children’s everyday life. This is especially true for those with chronic disease, e.g., asthmatic children. In recent decades, the number of patients suffering from asthma and other respiratory disorders has continuously increased not only in Hungary, but all over the world. According to a 2019 estimate, asthma affects 262 million people worldwide and causes 455,000 deaths [1]. In 2015, Hungary had over 290,000 registered asthmatic patients, reflecting a prevalence of 2.94%. According to a study by Molnár and colleagues, the prevalence of asthma among children aged 6–12 years is 6.5% in Budapest [2].

The goal of different therapeutic approaches is not only to treat the disease, but to improve patients’ quality of life. Studies examining well-being and quality of life started in the 1970s [3,4]. In Hungary, the quality of life of asthmatic patients has also received some attention [5,6]. However, asthma-related surveys specialized to childhood asthma are often associated with clinical trials [7,8,9]. Nevertheless, studies on the long-term consequences of childhood asthma on quality of life revealed reduced exercise habits in adults [10].

Data available in the literature on exercise in childhood asthma are conflicting [11]. Several studies have reported that children with asthma have lower levels of physical activity than their healthy peers [12,13,14,15], while other studies have shown no difference in physical activity between sick and healthy children [16].

The exercise habits of children with asthma are influenced by a variety of factors, including their own fears and experiences of being physically restricted by their illness, or the perceptions of their parents or family members about asthma and/or physical activity [17]. Evidence also suggests that better fitness levels and better cardiorespiratory status improve/decrease asthma symptoms. Nonetheless, using individualized exercise programs with adjusted intensities is just as important to achieve positive effects [11,18]. Furthermore, regular exercise also reduces obesity and osteoporosis, which are common complications of steroid treatments used in asthma [19]. Studies to date thus suggest that regular physical activity is a protective factor against the development of asthma [20].

In an overweight individual, intense physical exercise as running might trigger coughing and breathlessness, which, in children, is difficult to distinguish from asthma symptoms without taking objective lung function measurements [21]. In children with asthma, physical activity may also trigger these symptoms, leading to wheezing and breathlessness, which could result in the individual refraining from more intense exercise or physical activity. This, consequently, may be associated with a higher body mass index (BMI) in asthmatic children [22].

However, the association between higher BMI and asthma has not yet been clearly established. Magnus and colleagues [23], in a study of more than 24,000 children, found that accelerated weight gain from birth to 3 years of age positively influenced the development of asthma. Clarke and colleagues found significantly higher BMI values, standardized for body weight and age, in children with asthma who were also significantly more likely to be emotionally overeating than their healthy peers [24].

For the effective management of asthma in childhood, it is essential that parents support their children and do not add to their fears [25]. Research has shown that parents, their characteristics and attitudes, such as higher education of the mother, the father’s smoking, or the parents’ asthma, play a significant role in the development and control of childhood asthma [26,27]. Parents’ attitudes towards medication and anxiety about the disease were significantly associated with asthma control, and also influenced asthma outcomes through diet and exercise habits [28]. Therefore, the extent to which either the child or the parent believes that they can influence the disease through a healthy lifestyle will also affect its outcomes [29].

The study aims to demonstrate the extent to which the societal perception of a chronic illness as a hindrance to physical activity exists. Two aspects of this question were analyzed. The first was whether children with asthma are willing to engage in physical activity and whether this affects their social integration. The other was the extent to which parents inhibit or support their children to be active in sports. We were curious to see if any correlation could be detected between the severity of the disease and the child’s physical activity and the closely-related BMI.

We hypothesized that the physical activity of children with asthma is negatively influenced by their fear of performance and parental concern about worsening symptoms. We also hypothesized that there are no sex differences in the above findings. We further hypothesized that the more severe the disease, the less physical activity the child performs, and that this is associated with an altered BMI.

Our study fills a niche, in that no such study has been conducted in this disadvantaged region of Hungary, where the levels of education and per capita income are low, and unemployment and the prevalence of chronic diseases are high [30].

## 2. Materials and Methods

### 2.1. Study Design

We conducted our study among children with asthma at the Department of Pediatrics, University of Debrecen, Hungary in Spring 2019. We examined their physical activity habits, their favorite sports, and various barriers to physical activity using a structured questionnaire for data collection. The questionnaire was developed specifically for this study and consisted of the following sections: (1) socio-demographic characteristics, (2) physical activity, and (3) quality of life.

To assess the physical activity of the participants, we employed a short version of the International Physical Activity Questionnaire (IPAQ) [31,32] and incorporated additional specific questions on physical activity from a validated questionnaire utilized in our previous studies [33,34]. Parents were also asked about their children’s other daily activities, such as ‘Does your child attend physical education classes at school?’, ‘Does your child attend physiotherapy?’. The questionnaire was reviewed by 6 experts and tested by 10 parent volunteers. The children’s quality of life was assessed using questions from the Pediatric Asthma Quality of Life Questionnaire (PAQLQ) [35]. 

We asked 4 questions about socio-demographic data, followed by 8 questions related to sporting habits, in the form of multiple-choice answers. The questionnaire was answered by the parents, i.e., how often, what, where, and with whom the child likes to play sports, and what is the motivation to be physically active. One more question was asked about the reasons for not playing sport, where several answers could be ticked. This was followed by 11 statements about the relationship between physical activity and asthma, covering the benefits of physical activity, the appropriateness of physical activity for children with asthma, and the parents’ feelings about their child’s exercise, with responses given on a 5-point Likert-type scale: ‘strongly disagree’, ‘disagree’, ‘no opinion’, ‘agree’, and ‘strongly agree’.

The Asthma Control Test, translated into Hungarian, was the last part of the questionnaire, which is a validated questionnaire designed for individuals with asthma [36,37]. The test estimates both daytime and nighttime asthma symptoms, medication use, and the impact of asthma on daily activities over the past 4 weeks including data on respiratory function. 

All questions were answered by the parents of the children in this study during their visits for check-ups. The interviewers, who are assistants working at the investigation site, recruited parents of sick children. Upon being informed of the purpose and procedure of the study, the parents provided written consent to participate. 

In the study we also evaluated the following spirometry parameters: vital capacity (VC), forced expiratory volume in 1 s (FEV_1_), and forced expiratory flow at 25% to 75% (FEF_25-75_) using a Piston Pre-201 spirometer (Piston Medical Ltd., Budapest, Hungary). These measurements, together with height and body weight of the children, were measured by a pediatric pulmonologist during the children’s visit to the Department of Pediatrics for check-up. For the interpretation of the former data, we relied on the literature on respiratory function tests [38]. Asthma was classified as mild, moderate, or severe based on nocturnal symptoms, FEV_1_, and FEF_25-75_. It was considered mild if nocturnal symptoms occurred no more than 3–4 times per month, FEV_1_ was greater than 80% of the reference value, and FEF_25-75_ was reduced in no more than 20% compared to the reference value. We categorized asthma as severe when nocturnal symptoms occurred several times a week, FEV_1_ was less than 60% of the reference value, and FEF_25-75_ decreased by more than 30%. All other cases were considered moderate.

### 2.2. Participants

All parents who brought their children (*n* = 138) to the outpatient care were asked to participate; finally, 93 parents and their 93 children agreed to be involved in the survey. Extracurricular sport was defined as a continuous physical exercise that lasted at least 30 min. The average age of children was 12.6 ± 3.5 years (mean ± SD), 65 (69.9%) were boys, 28 (30.1%) were girls. In 2023, after the COVID-19 pandemic, 16 of the 93 children were still followed by the Department of Pediatrics, and those patients repeated the filling in of the same questionnaire and medical assessment.

The study adhered to the Declaration of Helsinki on Ethical Principles for Medical Research Involving Human Subjects and was approved by the Medical Research Council of Hungary (ETT-TUKEB: 24634-4/2018/EKU).

### 2.3. Data Analysis

The completed questionnaires were processed using EvaSys 8.2. software (VSL Inc., Szentendre, Hungary; http://www.vsl.hu). Responses were analyzed by sex. SPSS (Statistical Package for the Social Sciences) 29.0 software (SPSS Inc., Chicago, IL, USA) was used for statistical analysis. The normal distribution of the data was checked using Kolmogorov–Smirnov test and Shapiro–Wilk test. In the case of multiple responses, frequency was analyzed using multiple response frequencies and the correlation between groups was examined using multiple response crosstabs. The Pearson’s Chi-square test or Fischer’s exact test was used for comparing proportions. The significance of differences between groups was assessed using the Mann–Whitney–Wilcoxon test or the paired Student’s *t*-test. A *p* value < 0.05 was considered statistically significant.

## 3. Results

### 3.1. Socio-Economic Factors Influencing Sports Behavior

**Figure 1:**
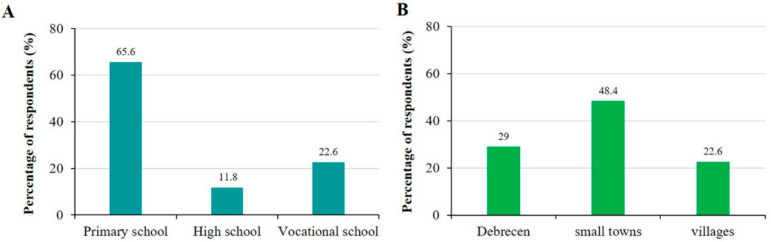
Demographic characteristics of children. (**A**) School attendance of the children. (**B**) Place of residence.

Close to one third of children studied in primary school, while the rest of them studied in different types of high schools (high school, technical college, technical school) (Figure 1A). Less than one third of the subjects lived in the county center Debrecen; almost half of them lived in different towns, while the rest lived in villages (Figure 1B). Analysis by residence showed no significant difference between proportion of those engaged in physical activity (55%, 46.7%, and 74.1%; in villages, towns, and Debrecen, respectively, *p* = 0.075), participation in PE classes (100%, 91.1%, and 92.3% in villages, towns, and Debrecen, respectively, *p* = 0.397) and physiotherapy (9.5%, 8.9%, and 19.2% in villages, towns, and Debrecen, respectively, *p* = 0.401). We investigated the relationship between choice of sport and place of residence, but did not find a significant association. In terms of reasons for physical inactivity, we observed that inactivity due to lack of facilities was significantly more common among children living in villages (*p* = 0.028; Chi-square test).

In total, 48.4% of the families reported a good standard of living, including financial savings, and 45.1% could cover all their expenditures without being able to save money. Only 2.2% of the subjects reported poor living circumstances and suffered from poverty in their everyday lives. Parents who believe that physical activity is important for a child with asthma, and that exercise improves asthma, reported better financial status, while parents who said that they live day to day were more likely to think that physical activity makes asthma worse (*p* = 0.050; barely significant).

### 3.2. Physical and Health Status of Participating Children

The BMI of children was very different, ranging between 12.3 and 37.4 kg/m^2^. Based on their BMI, 24 children were considered overweight (relative BMI > 75%), while 13 of them were underweight (relative BMI < 10%). However, the mean value of BMI normalized to their ages was 100 ± 19%, thus corresponding to the values of healthy children at the same age. We also examined the relationship between children’s BMI status and their physical activity, but no significant difference was found between the groups (*p* = 0.398, Chi-square test). The percentages of children doing and not doing physical activity were almost the same in all groups. Furthermore, the analysis of BMI status also revealed no significant difference between their participation rates in physical education (91.2%, 95.7%, 85.7%, and 100%; underweight, normal, overweight and obese, respectively, *p* = 0.698) and physiotherapy (17.1%, 10.9%, 0%, and 0%; underweight, normal, overweight and obese, respectively, *p* = 0.548). When comparing the BMI status of children preferring different sports, we found significant differences for cycling (*p* = 0.046) and gymnastics (*p* = 0.016). For both, underweight children chose these sports more often than their overweight counterparts. When examining the association between reasons for physical inactivity and BMI status, we observed that significantly more children with normal BMI did not play sports due to lack of time (*p* = 0.008). Moreover, the analysis showed that children with asthma who were overweight are more likely to be recommended by their doctor not to be physically active (*p* = 0.007).

Conclusions were similar upon examining respiratory parameters (vital capacity, VC; forced expiratory volume in 1 s, FEV_1_, and forced expiratory flow at 25% to 75%, FEF_25-75_). Minimum and maximum values of VC, FEV_1_, and FEF_25-75_ normalized to the expected age-related values which were 24 and 123%, 39.6 and 140%, 22.7 and 128%, respectively. As expected, strong correlation was detected between the values of FEV_1_, and FEF_25-75_ (r^2^ = 0.524). Also, the mean value of FEF_25-75_ (74.4 ± 21.6%) was significantly lower compared to the expected value at that age. However, there was no correlation neither between BMI and FEV_1_ (r^2^ = 0.0004), nor between BMI and FEF_25-75_ (r^2^ = 0.0002), respectively.

### 3.3. Sporting Habits of Children with Asthma

A total of 56.5% of the children reported doing regular physical activity besides PE classes, 46.3% performed physical exercise once or twice a week, and 38.9% three to four times a week, while 11.1% of them exercised at least five times a week.

**Figure 2:**
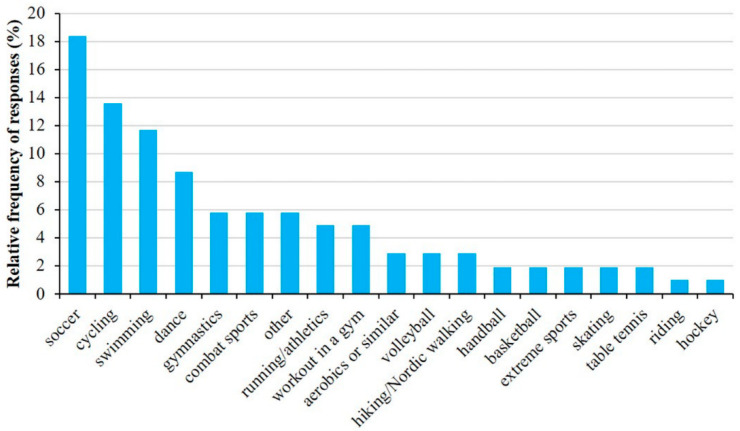
Relative frequency of types of sport among children with asthma. Data are presented for all respondents, and they were allowed to select more than one response.

The most common forms of physical activities (Figure 2) were soccer (18.4%), cycling (13.6%), swimming (11.7%), and dancing (8.7%). Children usually exercised in school (26.9%) as part of their afternoon activities or in different sport clubs (21.5%). Some exercised at home or chose outdoor activities.

**Figure 3:**
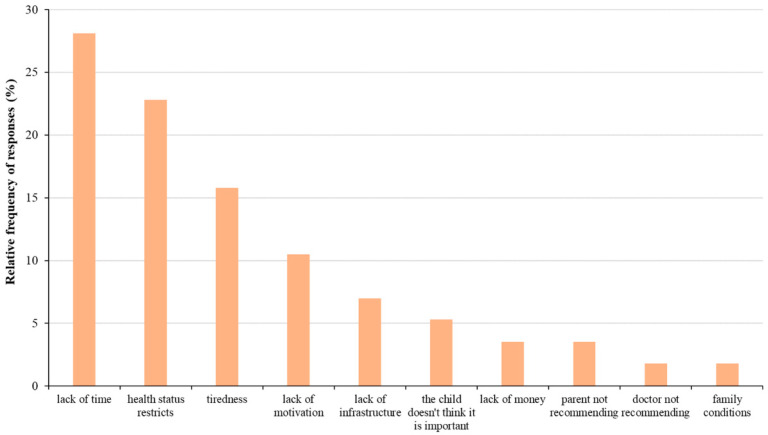
Relative frequency of reasons for not playing sports among asthmatic children. Respondents were allowed to choose more than one answer.

However, even those who did not exercise regularly performed frequent physical activity such as cycling or walking. This is likely because the majority of the subjects lived in small towns where cycling or walking were the prevailing modes of transportation. It is important to note that, except for 6.6%, almost all children participated in PE classes. In correlation to that, the percentage of children going to physiotherapy was very low, only 12%. However, the percentage of children who did not exercise due to their health status was very high (22.8%) among those who did not perform regular physical activity. Lack of time (28.10%), tiredness (15.8%), and lack of motivation (10.5%) were the other most common reasons for physical inactivity (Figure 3).

**Figure 4:**
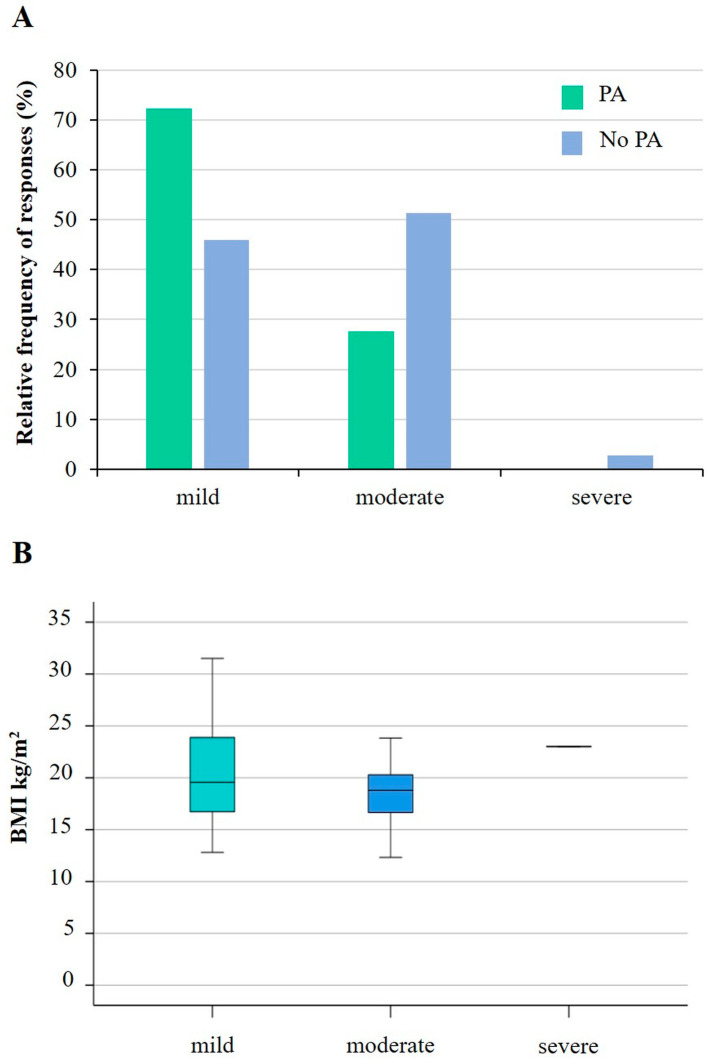
The association between asthma status and physical activity (**A**) and BMI (**B**) in children with asthma. While a significantly lower number of children participate in physical activity as the severity of asthma increases (*p* = 0.035; Chi-square test), a similar conclusion for BMI could not be drawn (*p* = 0.138; Chi-square test). PA: physical activity; No PA: no physical activity; BMI: body mass index.

Figure 4 illustrates the relationships between asthma severity, physical activity, and body mass index. While the number of children doing physical activity decreases significantly with increasing asthma severity (*p* = 0.035), there was no correlation (r^2^ = 0.0269) with BMI.

**Figure 5:**
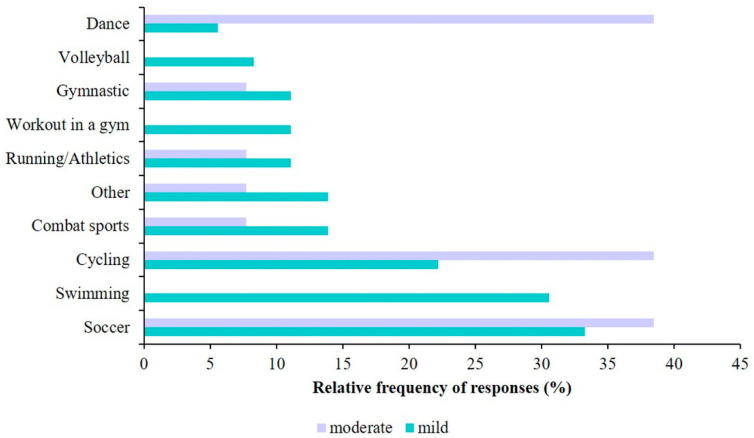
Top ten chosen sports for children with asthma.

In terms of different types of sports, Figure 5 presents the most common activities performed by children with mild and moderate asthma. A significant difference between the two groups was only found for swimming (*p* = 0.018; Chi-square test). Regarding participation in PE classes and asthma status, significantly more children with mild (62.3%) and moderate asthma (37.7%) participated in PE classes, while children with severe asthma (16.7%) typically did not attend PE classes at school (*p* = 0.002; Chi-square test). However, we did not find a significant difference between participation in physiotherapy and asthma status (*p* = 0.140; Chi-square test). Analyzing the association between the reasons for physical inactivity and asthma status, we observed that the only significant difference among the different groups was in physical inactivity due to health status (*p* = 0.023).

### 3.4. Parents’ Opinion on the Physical Activity of Their Children

A total of 77.2% of parents found the role of physical activity important for asthmatic children. However, only 56.5% said that physical activity could improve the disease.

**Figure 6:**
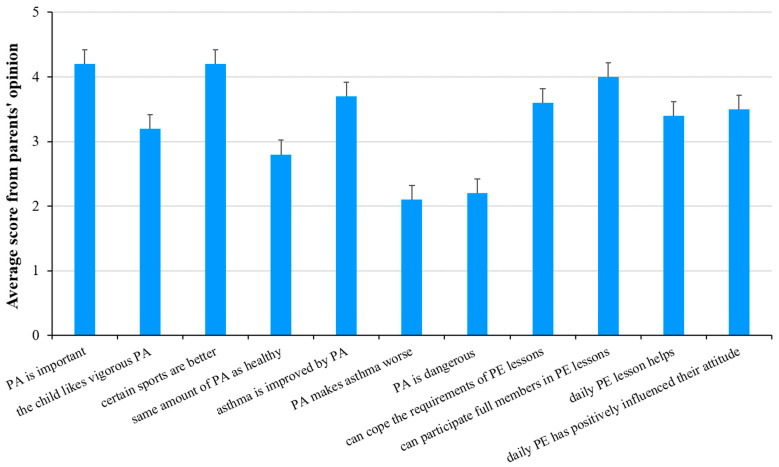
Parents’ opinion, on a scale of 1 to 5, on the relation of physical activity to asthma. Mean ± SD is shown.

A total of 56.1% of parents believed that their children could meet all the requirements of PE classes. Similarly, the percentage of parents who believed that their children could participate in PE classes just like their healthy peers was about the same (Figure 6).

Differences in parents’ responses for those whose children exercised regularly, versus those whose did not, was also considered (Table 1). Those parents whose children exercised regularly believed that physical activity was important for asthmatic children, improved the asthma, and that their children could participate in PE classes with the group just like healthy children (Table 1). In contrast, the latter group’s judgement on these points was significantly worse (3.87, 3.33, and 3.49, respectively). Rating the significance of everyday exercise was also different among the two groups of parents. Parents of those children who exercised regularly said that daily PE classes had improved the health status of their children and positively affected their attitude toward physical activity. On the other hand, parents of those children who did not exercise regularly thought that the effect of daily PE class was negligible (2.95 and 3.05, respectively). The differences between the parents’ responses for groups of children who regularly participate in physical activity and those who do not are shown in Table 1. Significant *p*-values are indicated in bold and italic, and *p* values < 0.05 were considered statistically significant.

### 3.5. Impact of Illness on Children’s Quality of Life

Close to half of the parents (49.5%) reported that their child indicated no asthma-related dyspnoea during daily activities in the last four weeks. A little over one third (37.6%) suffered from that once or twice a week, while only one child (1.1%) suffered more than once a day.

**Figure 7:**
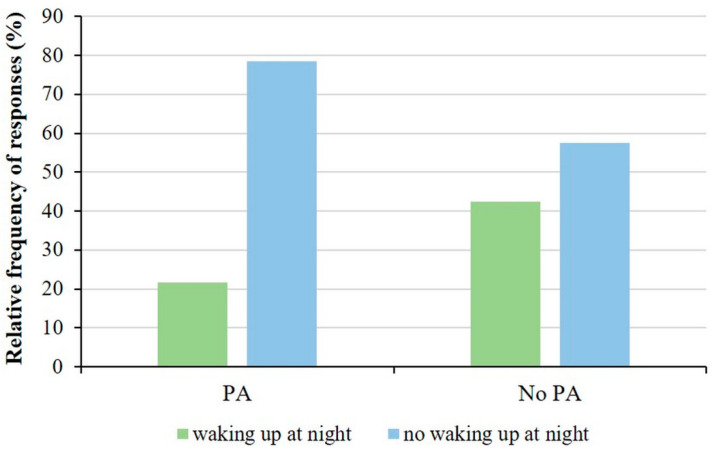
Association with the physical activity and sleep disturbances in children with asthma. Significantly higher number of children doing physical activity who did not experience sleep disturbance due to asthma symptoms (*p* = 0.041; Fisher’s exact test).

Studying the association between physical activity and sleep disturbances, we observed that 21.6% of children who participated in sports activities woke up at night, or earlier than their usual wake-up time in the morning, with asthma symptoms, while 78.4% did not wake up. In contrast, 42.5% of children who do not play sport had sleep-disrupting asthma symptoms. Reconsidering the options given in the questionnaire, we created two groups, children who woke up at night for asthma symptoms and children who did not wake up. Thus, when performing the analysis, we observed a significant difference between the physical activity group and the frequency of asthma symptoms that cause sleep disturbances (*p* = 0.041; Fisher’s exact test, Figure 7).

When analyzing the association between physical activity and asthma attacks, we did not find a significant correlation. However, only 19.2% of children who were physically active had used the asthma medication, while all the others had not used it at all in the 4 weeks before completing the questionnaire. In contrast, 40% of the children who did not do sports used their medicine in the given period.

### 3.6. Sex-Based Analysis of Physical Activity of Children with Asthma

Separate analysis of the answers from boys and girls revealed no significant difference between the rate of their physical activity (50.0% and 59.4% for girls and boys, respectively), participation in PE classes (96.3% and 92.2% for girls and boys, respectively) and in attending physiotherapy (14.8% and 10.8% for girls and boys, respectively). However, similarly to healthy children at the same age [34,39,40], their choice of exercise type differed significantly. Girls mostly preferred dancing (25%) and gym (10.7%), while boys rather chose soccer (26.2%) and cycling (20%). The relative frequency for physical activity type by sex are summarized in Table 2 and significant differences are indicated in bold and italic.

The parents of boys and girls agreed that physical activity was important for asthmatic children (4.22 and 4.18 for girls and boys, respectively), and that certain sports were better for them (4.11 and 4.20 for girls and boys, respectively). However, judgement of physical activity was very different among the parents. Parents who had boys thought that physical activity could improve their sons’ health status (3.8) and they could participate in PE classes just like healthy children (4.17). However, this was not obvious for parents who had girls (3.32 and 3.74, respectively). Table 3 summarizes the responses, and significant differences are indicated in bold and italic. Parents of girls are significantly less likely to agree (2.33) with the statement that children with asthma can do the same amount of physical activity as their healthy peers (*p* = 0.023). Parents of boys disagree that physical activity is dangerous for children with asthma (2.05), while parents of girls are more neutral (2.68), and the difference is statistically significant (*p* = 0.020). Furthermore, parents who had boys believed that the introduction of daily physical education had improved their child’s health (3.54, significantly different from those with girls; *p* = 0.042, Table 3). The relationship between the reasons for physical inactivity and sex were also analyzed, but no significant association was found.

### 3.7. Results of Follow-Up Examinations

In the cases of 16 children, it was possible to compare the values of asthma-related parameters measured in 2019 with those identified in 2023. We examined BMI trends and observed a significant increase. As expected, absolute values of VC, FEV_1_, and FEF_25-75_ increased from 2019 to 2023 as the children got older. Comparing the relative values of VC and FEV_1_, on average, higher values were observed in 2023, but the change was not significant. However, when comparing the FEF_25-75_ parameters, a reduced average value was observed in 2023, although the difference was again not significant. Table 4 reports results for the comparison of the asthma-related variables in the study group and significant differences are indicated in bold and italic.

## 4. Discussion

In the past 50 years, several studies analyzed the effect of physical activity on childhood asthma [11,41,42,43,44,45,46,47]. Many studies suggested regular physical activity for asthmatic children [48,49], since it could improve both their physical conditions, their social interactions, and their development as well [50]. Several studies proved that obesity aggravated the severity of asthma and attenuated quality of life [45,51,52,53], strongly suggesting proper diet and regular physical activity [54]. Scientists stated that both competitive sport and physical activity were important to carry out a proper lifestyle that is indispensable not only for adults and healthy people, but asthmatic children as well [54].

### 4.1. General Findings

In this study, we examined the exercise habits of 93 asthmatic children, as well as their parents’ relation to their physical activity. The results confirmed our hypothesis that asthma, i.e., being aware of the disease, negatively influences children’s physical activity. Our assumption that fear and parental attitudes underlie this was also verified. However, although we found no sex differences in children’s physical activity; parents of girls and boys differed in their approach towards the physical activity of their children, with the former being less supportive. Our data showed a clear correlation between asthma severity and physical activity, whereas no such correlation with BMI was observed.

### 4.2. Physical Activity

Based on our results, we concluded that the percentage of those who performed regular physical activity was smaller for asthmatic children compared to their healthy peers. While 90% of teenagers from northeast Hungary exercised regularly, as demonstrated in our earlier study [40], it was less than 60% for those asthmatic children who participated in this study. When examining the reasons for not engaging in physical activity, the proportion of non-participants citing “health status restricts” as a reason for non-participation was found to be higher among children with asthma (22.8%), compared to the general adolescent population (15.9%) [39,40]. In their study, Winn and colleagues [55] also found that adolescent children with asthma performed less physical activity than their healthy peers. Wanrooij and colleagues [11], in a systematic review, likewise described that there is a large body of evidence suggesting that children with asthma are less physically active than their healthy peers. However, they also pointed out that there are several other studies suggesting that such differences cannot observed. Similar to our results, Mackintosh and colleagues [56], in their systematic review and meta-analysis, showed no difference between boys and girls with asthma in terms of the proportion who regularly engaged in physical activity.

On the other hand, similar to prior observations on healthy children, the place of living greatly affected the significance of physical activity [39]. While children in small towns and villages (supposedly due to the lack of proper infrastructure [40]) exercised mostly in school or outdoors (38.1 and 19.0%, respectively), children in county centers exercised in sport clubs or sport facilities (33.3 and 22.2%, respectively). As team sports are often the easiest ways to get asthmatic children involved into physical activities [57], it is thus important to emphasize that providing proper infrastructural conditions could have a significant impact on their commitment to being physically active.

We also observed that the severity of asthma was related to fewer children engaging in physical activity, which is in line with the literature [58,59]. However, no association was found between children’s BMI status and physical activity. Although early childhood obesity may predispose asthma, no clear association between the presence of asthma and child BMI has been demonstrated so far [58,59,60]. Further studies are thus needed to explore the possible association between asthma severity and BMI and the underlying reasons.

### 4.3. Parental Attitudes

One important aspect of our examination was the parents’ attitude toward the physical activity of their asthmatic children. Interestingly, those parents whose asthmatic children exercised regularly had a more positive attitude toward sports and daily PE classes than those parents whose children did not. Further studies should explore whether this was the consequence of the positive experiences the former, or the fear of worsening the symptoms of the latter, or both. 

Another conclusion from our data was that parents with boys supported exercise more than parents with girls, which could be due to the fact that parents with daughters had a tendency to take less risks. Similarly, Beets and colleagues found gender differences in family support for physical activity [60]. Some studies indicated that boys receive significantly more family support for their physical activity than girls do [61]. Recent reports concluded that the main limiting factor for asthma patients regarding physical activity is fear of asthma attacks [57,62]. Therefore, parental support, including verbal encouragement, financial contributions, and time investment, is crucial for an asthmatic child’s physical activity, which raises the need for further education of these parents.

### 4.4. Quality of Life and Physical Activity

The worsening of the symptoms of bronchial asthma during nighttime is a well-known phenomenon and is associated with sleep disturbances [63]. Our results indicate that children with asthma experience better sleep quality when they engage in some form of active exercise during the day. Nnodum and colleagues also identified a significant association between physical activity and both daytime and nighttime symptoms [64]. In line with the data presented here, the improvement in quality of life of asthmatic children was confirmed in a systematic review by Wanrooij and colleagues [11]. Furthermore, a reduction in the need for daily medication was reported by the children who were regularly engaged in physical activity, again in agreement with previous reports [11]. Moreover, among physically inactive children with asthma, others also have reported poorer quality of life [65].

### 4.5. Limitations

There are certain limitations of the study. Although we tried to include all patients with asthma at the Department of Pediatrics, only 93 parents agreed to participate in the study. A group of 20 children were also given a wristband (Xiaomi Mi Band 2, Xiaomi Corporation, Beijing, China; readout: steps in every minute) to monitor daily physical activity. However, only nine of these children wore the wristband continuously long enough for the data to be properly evaluated. These data could not be included in the analysis as no meaningful conclusions could be drawn. We also aimed to investigate the impact of the COVID-19 pandemic on these children, we thus conducted a follow-up survey in 2023. Unfortunately, we were only able to repeat the survey for 16 out of the 93 children.

The exercise habits of children with asthma are influenced by a number of factors, many of which were not examined here. These include, but are not limited to, whether or not the child’s parents or other family members have asthma, other characteristics of the parents, such as education, employment, leisure time, or whether they exercise regularly. We also did not register the gender of the parents. We asked the parents’ opinions about their children, but we did not measure the children’s fitness levels directly. Investigating the above could be an important aspect of future research.

The study population was from the outpatients of the pediatric department, thus only a few reported severe or untreated asthma status. Due to the regional nature of the patient care and the importance of environmental factors, the application of our results to other populations should be done with caution. Moreover, the number of cases did not allow for age-group analysis.

Finally, although parts of the questionnaire used in this study were taken from validated questionnaires, the composed list of questions was first used here. To ensure its validity, we asked experts in the field to review the questions and ten volunteer parents to fill it in before starting the actual survey.

## 5. Conclusions

Our everyday choices, including our health behaviors, are influenced by both our positive experiences and our fears. To ensure that the proportion of children with asthma who exercise regularly is not lower than in their healthy peers, it is important that both they and their parents are well informed about the benefits of exercise. Although the first study showing that physical activity improves the health of asthma patients was published several decades ago, there are still a significant number of parents and children who do not think or experience this, and, as a result, the proportion of children with asthma who exercise is lower than it should be.

Therefore, we have to strive to prove that asthmatic children should enjoy the benefits of regular physical activity. Besides providing proper infrastructural conditions, choices of appropriate sports and professional support are also necessary.

It is, therefore, necessary that family doctors and pediatric pulmonologists educate parents and children, and inform school PE teachers, about the exercises that children with asthma can do and the importance of personalized exercise intensity. It is important that children, and therefore their parents, have positive experiences in PE lessons at school or when trying out a sport.

Future research should investigate the sporting habits of children with asthma in the context of family, school, and home infrastructural environments, in order to have these influences analyzed in a large sample. It would seem necessary to carry out analyses by age group, as the proportion of the general population who regularly take part in physical activity also varies at different stages of life.

As there are conflicting data in the literature on the development and controllability of asthma in relation to BMI, further measurements are needed in this direction. Detailed exploration of other environmental factors should also continue.

## Figures and Tables

**Table 1:** Parents’ opinions * on the relationship between their children’s physical activity and asthma.

Statement	Mean (SD)		*p*-Value ^a^
	Physically Active Children	Physically Inactive Children	
Physical activity is important for asthmatic children.	4.44 (0.80)	3.87 (1.08)	***0.006***
The child likes vigorous physical activity.	3.63 (1.28)	2.56 (1.05)	<***0.001***
Certain sports are better for children with asthma.	4.33 (0.84)	4.00 (0.92)	0.062
Children with asthma can do the same amount of physical activity as their non-asthmatic peers.	2.98 (1.38)	2.44 (1.17)	0.071
Asthma is improved by physical activity.	3.87 (1.07)	3.33 (1.20)	***0.033***
Physical activity makes asthma worse.	1.98 (1.09)	2.35 (1.19)	0.139
Physical activity is dangerous for children with asthma.	2.06 (1.02)	2.46 (1.27)	0.160
My child feels that he/she can cope the requirements of PE lessons.	3.98 (1.53)	3.08 (1.42)	***0.002***
My child feels that he/she can participate fully in group sports activities in PE lessons.	4.45 (1.10)	3.49 (1.30)	<***0.001***
My child’s health has been improved by the introduction of daily physical education.	3.63 (1.25)	2.95 (1.26)	***0.010***
My child’s attitude to physical activity has been positively influenced by the introduction of daily PE.	3.87 (1.30)	3.05 (1.30)	***0.002***

* 1: Fully disagree, 5: Fully agree; ^a^ Mann–Whitney–Wilcoxon test. Significant *p*-values are indicated in bold and italic (*p* < 0.05).

**Table 2:** Relative frequency for physical activity type by sex.

Type	Girls (%)	Boys (%)	*p*-Value ^a^
**Dance**	25	3.1	***0.001***
Gymnastics	10.7	4.6	0.272
Swimming	10.7	13.8	0.679
Aerobics or similar	7.1	1.5	0.161
**Soccer**	7.1	26.2	***0.037***
**Handball**	7.1	0	***0.029***
Volleyball	7.1	1.5	0.161
Running/Athletics	3.6	6.2	0.613
**Cycling**	3.6	20	***0.042***
Riding	3.6	0	0.126
Skating	3.6	1.5	0.535
Other	3.6	7.7	0.458
Workout in a gym	0	7.7	0.131
Basketball	0	3.1	0.348
Hiking/Nordic walking	0	4.6	0.248
Combat sports	0	9.2	0.096
Extreme sports	0	3.1	0.348
Hockey	0	1.5	0.509
Table tennis	0	3.1	0.348

^a^ Pearson’s Chi-squared test; Significant *p*-values are indicated in bold and italic.

**Table 3:** Results of parents’ opinions * for girls (*n* = 28) and boys (*n* = 65).

Statement	Mean (SD)		*p*-Value ^a^
	Girls	Boys	
Physical activity is important for asthmatic children.	4.22 (0.97)	4.18 (0.97)	0.864
The child likes vigorous physical activity.	2.81 (1.33)	3.34 (1.25)	0.091
Certain sports are better for children with asthma.	4.11 (0.89)	4.20 (0.89)	0.592
Children with asthma can do the same amount of physical activity as their non-asthmatic peers.	2.33 (1.39)	2.94 (1.25)	***0.023***
Asthma is improved by physical activity.	3.32 (1.16)	3.80 (1.13)	0.053
Physical activity makes asthma worse.	2.32 (1.09)	2.05 (1.17)	0.250
Physical activity is dangerous for children with asthma.	2.68 (1.25)	2.05 (1.05)	***0.020***
My child feels can cope the requirements of PE lessons.	3.36 (1.55)	3.70 (1.54)	0.225
My child feels that he/she can participate fully in group sports activities in PE lessons.	3.74 (1.46)	4.17 (1.18)	0.177
My child’s health has been improved by the introduction of daily physical education.	2.93 (1.30)	3.54 (1.26)	***0.042***
My child’s attitude to physical activity has been positively influenced by the introduction of daily PE.	3.29 (1.41)	3.63 (1.33)	0.257

* 1: Fully disagree, 5: Fully agree; ^a^ Mann–Whitney–Wilcoxon test; significant *p*-values are indicated in bold and italic (*p* < 0.05).

**Table 4:** Comparison of asthma-related parameters.

Variables		Mean (SD)		t-Value	*p*-Value ^a^
		2019	2023		
BMI	(kg/m^2^)	18.46 (3.95)	21.94 (4.14)	−3.752	***0.002***
VC	(L)	2.81 (1.16)	3.21 (1.18)	−2.268	***0.039***
	(%)	88.66 (16.43)	93.91 (20.29)	−1.117	0.282
FEV_1_	(L)	2.39 (0.99)	2.72 (0.99)	−2.246	***0.040***
	(%)	87.69 (13.20)	88.59 (19.84)	−0.208	0.838
FEF_25-75_	(L/s)	3.21 (1.12)	3.55 (1.19)	−2.400	***0.030***
	(%)	76.82 (18.82)	74.56 (22.18)	0.549	0.591

^a^ Student’s *t*-test; BMI: body mass index, VC: vital capacity, FEV_1_: forced expiratory volume at 1 s, FEF_25-75_: forced expiratory flow at 25% to 75%. VC, FEV_1_, and FEF_25-75_ values are normalized to the expected age-related values. Significant *p*-values are indicated in bold and italic (*p* < 0.05).

## Data Availability

The data obtained during this study are available from the corresponding author upon reasonable request.
